# The Roseto Study: Selection Bias Versus Social Support

**DOI:** 10.7759/cureus.113024

**Published:** 2026-07-20

**Authors:** Samrachana Adhikari, Olugbenga G Ogedegbe, Orrin Devinsky

**Affiliations:** 1 Department of Population Health, NYU Grossman School of Medicine, New York, USA; 2 Institute for Excellence in Health Equity, NYU Grossman School of Medicine, New York, USA; 3 Department of Neurology, NYU Grossman School of Medicine, New York, USA

**Keywords:** diet, health, obesity, saturated fat, social support

## Abstract

Background

A landmark study of 1,600 Italian-Americans in Roseto, PA, challenged the prevailing view that high saturated fat intake was a major cause of myocardial infarction (MI). Despite similar rates of cigarette smoking and obesity, and even higher levels of saturated fat consumption compared to neighboring towns, Rosetans experienced far lower MI death rates. More than 50 years later, it remains uncertain whether Roseto’s residents had better heart health than the average American and, if so, what protective factors may have been responsible.

Methodology

We compared MI deaths in Roseto and neighboring towns to the contemporaneous Framingham Heart Study cohort matched for age and sex.

Results

We found no evidence that MI deaths were lower in Roseto, PA, than in Framingham, MA when controlling for age and sex. While the role of social support in health has been established in subsequent studies, methodological issues, confounding factors, and biases challenge the validity of the Roseto study.

Conclusions

The dramatically lower MI and MI mortality rates among males in Roseto reflect biases in sampling and comparison populations, which also impacted the contrasting Diet-Heart Hypothesis that saturated fats cause heart disease. Although social support enhances health outcomes, the Roseto study neither supported nor refuted this connection.

## Introduction

American men faced an apparent epidemic of myocardial infarction (MI) deaths in the 1950s. In 1964, Stewart Wolf and colleagues reported that men in Roseto, PA, rarely died from MI [1]. This Italian-American enclave of 1,600 people had similar rates of cigarette smoking and obesity and higher saturated fat consumption than did most of America. Wolf et al. interviewed and examined every Rosetan adult and many in the neighboring towns of Bangor, Nazareth, Stroudsburg, and East Stroudsburg. Suspected risk factors were similar among residents in these towns. They argued that genetic factors were unlikely, as other Italian-Americans and Rosetans who migrated out to other towns had higher MI rates than the Italian-Americans in Roseto.

The objective of this study is to compare the sociocultural and environmental factors potentially contributing to the lower incidence of MI observed in Roseto, PA, compared to patterns in neighboring towns and the Framingham Heart Study (FHS) cohort. We compared how MI death rates in Roseto and neighboring Pennsylvania towns compared to those in Framingham, MA, ascertained as part of the prospective FHS. We assessed MI mortality rates in Roseto, neighboring towns, and the FHS. In addition, we explored the potential roles of social support, diet, and other factors.

The Roseto study challenged the prevailing consensus that saturated fat consumption was a risk factor for MI. By 1961, FHS identified older age, male sex, hypertension, hypercholesterolemia, and cigarette smoking as heart disease risk factors [2]. The “Diet-Heart Hypothesis” [3], introduced by University of Minnesota physiology professor Ancel Keys, posited that rising 20th-century fat consumption, especially saturated fat from animal sources, caused elevated cholesterol levels [3]. High cholesterol levels correlated with heart disease in the FHS and between nations (e.g., the United States and England versus Italy and Greece) [3,4].

The Roseto study emerged during a heart disease epidemic and a changing American diet. The role of genetics, dietary factors, and stress remained controversial. However, in 1961, the American Heart Association (AHA) recommended “The reduction or control of fat consumption under medical supervision, with reasonable substitution of poly-unsaturated for saturated fats, is recommended as a possible means of preventing atherosclerosis and decreasing the risk of heart attacks and strokes” [5]. Discoveries in the early 1960s about the role of refined carbohydrates in elevating triglycerides, small dense atherogenic low-density lipoprotein (LDL) particles, lowering high-density lipoprotein (HDL), and causing insulin resistance and metabolic syndrome [6] had little influence on dietary recommendations. Reducing total fats and saturated fats remained the primary dietary intervention recommended by the AHA to prevent heart disease [7], although the directors of the National Institutes of Health (NIH) and National Heart, Lung, and Blood Institute (NHLBI) recommended a low-carbohydrate diet to treat the most common form of hyperlipidemia in the United States. [8] In Roseto, Wolf et al. focused their etiologic lens not on diet but on the community’s fabric of social interactions, resonating on an intuitive chord while challenging the dietary orthodoxy.

Wolf et al. observed that the most striking feature of Roseto was its strong sense of community. Residents were described as living in a single social class, leading simple lives, warm and hospitable, with virtually no crime, and offering mutual support to one another. [1] They later highlighted the “Old World values and customs” [9] and their “cohesive social structure” [10] as key factors contributing to their good health. Rosetans’ good health is attributed to social support and neighborhood cohesion, helping launch the social-support health discipline [1,9,10]. This observation was based on informal observation of social interactions, which were not systematically quantified in Roseto or neighboring towns. Social relationships can support cardiac, mental, and other positive health outcomes across populations and settings [11]. The Roseto Effect faded from lay and medical awareness as support for the Diet-Heart Hypothesis transitioned from one among many hypotheses to the dominant, politically correct viewpoint. The Roseto story briefly re-entered the mainstream [12] with Malcolm Gladwell’s book Outliers (2002): “In Roseto, virtually no one under 55… showed any signs of heart disease. For men over 65, the death rate from heart disease in Roseto was roughly half that of the United States…. The Rosetans were healthy because of where they were from, because of the world they had created.”

By contrast, Keys proposed that saturated fats caused heart disease, relying on: (1) feeding studies showed saturated fats increased total serum cholesterol, 2) population-based studies correlated total cholesterol consumption with heart disease risk, and (3) international comparisons showing that populations with high saturated fat consumption had higher cholesterol levels and heart disease rates [3,4]. Keys criticized the Roseto study because the heart disease rate was not lower than the United States average, and Keys recognized the potential diagnostic and selection biases [3]. Further, some older Rosetans were born in Italy, and their childhood and young adult diets likely differed from their diet in America. These valid criticisms are also applicable to his Diet-Heart Hypothesis.

Were Rosetan men healthier than contemporary American men? Only selected data summaries from Roseto and neighboring Bangor, Nazareth, Stroudsburg, and East Stroudsburg populations were published [1]. The original paper detailed the age, sex, weight, and height of Rosetans and compared them to either neighboring towns or average Americans, not both. Deaths due to MI and other cardiac causes in Roseto and neighboring towns were reported, but the only diet and cholesterol levels were reported for Rosetans, introducing potential confounding bias. No data was provided to support the contention that Rosetan men who moved out died at relatively young ages from MI. Finally, data on the number of deaths due to cancer and other causes were not provided. With these limitations of the original paper, we revisit the Roseto study and explore five potential explanations for the “heart health” of Rosetans.

## Materials and methods

Comparison population

The FHS was the only US cohort with prospective mortality and cardiovascular disease (CVD) mortality data concurrent in time with the Roseto study. The FHS began in 1948 under the National Heart Institute (now NHLBI) to identify common factors or characteristics that contribute to CVD. Three generations have been enrolled; the original Cohort 1 comprised 5,209 men and women between the ages of 30 and 62 years old living in Framingham, MA, who were free of CVD symptoms or disease at study onset.

Statistical analysis

We first summarized reported MI mortality rates in Roseto, PA, from the Roseto Study [1,9,10] and in Framingham, MA, from the FHS. Next, we conducted a simulation study to investigate the divergence in observed and expected number of deaths in Roseto, when the underlying probability of MI death was similar to FHS. To simulate the expected number of deaths, we generated counts from a binomial distribution with underlying n corresponding to the number of people in each age group in Roseto in 1950 (extracted from the published paper) [13], and the probability was corresponding to the age-specific rate in FHS from 1955 to 1964 among the original FHS cohort. We repeated this 1,000 times for each age group and computed the corresponding 95% confidence interval from the distribution of the expected number of deaths. We finally assessed whether the observed number of deaths in Roseto was within the 95% confidence interval of the expected number. Statistical software R was used for analysis.

## Results

Part 1: simulated comparison of Roseto and neighboring towns to the FHS

First, age- and sex-specific MI mortality rates were computed from 1955 to 1964 among the original FHS cohort (2,294 men and 2,785 women, aged 28 to 74 years, recruited in 1948-1949) as FHS Cohort 1. We then simulated the expected number of deaths using a binomial process with the underlying FHS rate and population size from Roseto in 1950. The number of expected deaths was computed for age groups <35, 35-44, 45-54, 55-64, and 65+ years. Within each age group, deaths were repeatedly sampled 1,000 times from the underlying binomial distribution. Age-specific mean number of deaths, along with 95% confidence intervals, were compared with the observed number of deaths from MI in Roseto from 1955 to 1964. If the observed deaths were within the 95% confidence interval of the expected deaths, then the observed number in Roseto was similar to FHS.

**Figure 1:**
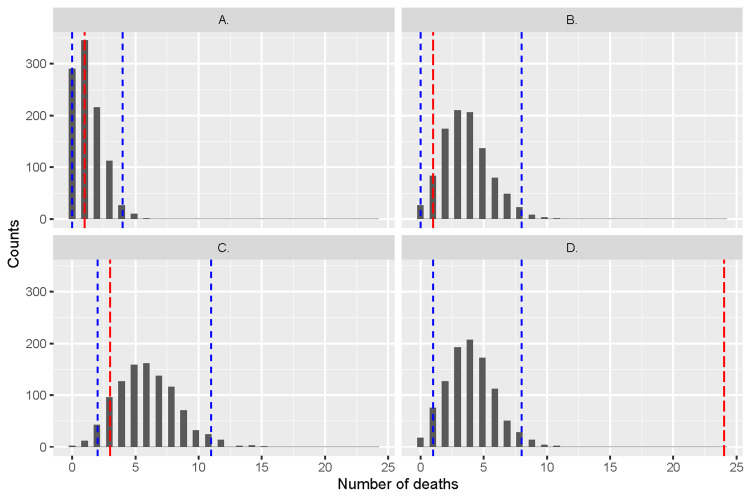
Role of chance and effect of low sample size. Histograms representing the distribution of the simulated expected number of deaths for Roseto when the underlying probability of CVD death is similar to that of the Framingham Heart Study. The red line represents the observed count. Blue lines represent the lower 2.5 and upper 97.5 percentiles of the simulated counts. (A) Simulated number of deaths for the age group 35-44 years. (B) Simulated number of deaths for the age group 45-54 years. (C) Simulated number of deaths for the age group 55-64 years. (D) Simulated number of deaths for those aged 65 and older.

Table 1 summarizes age- and sex-specific deaths per 1,000 population in FHS and Roseto. Figure 1 displays the simulated counts distribution in a histogram. The observed deaths in Roseto were within the 95% confidence interval of simulated deaths in all age groups except for the 65+ group, where it was higher than expected in Roseto. Therefore, observed deaths in Roseto were within the expected range compared to the FHS mortality rate.

**Table 1:** Summary of age- and sex-specific deaths per 1,000 population in FHS and Roseto. FHS = Framingham Heart Study

			Age group (years)			
Cohort	Year	Sex	35–44	45–54	55–64	65+
FHS	1955–1964	Male	5.6	35.2	81.5	31.5
		Female	2.5	7.6	20.2	32.8
		Total	4.3	23.5	55.6	32.1
	1965–1974	Male	0	11.7	42.9	87.1
		Female	0	4.2	19.8	68
		Total	0	8.1	31.9	78
Roseto study	1955–1964	Male	8.3	9.8	29	211.3
		Female	0	0	15.9	120
		Total	8.3	9.8	22.5	165.7
	1965–1974	Male	33.3	55	92.8	268.7
		Female	0	0	15.9	181.8
		Total	33.3	55	54.4	225.3

Part 2: examination of potential alternative explanations

Law of Small Numbers and Chance

Kahneman and Tversky postulated the law as a cognitive bias whereby humans inappropriately extrapolate from small groups to large groups. Small segments of a large population may not reflect the larger cohort. For example, health outcomes of a small, low socioeconomic population cannot be extrapolated to a larger, more affluent population. We, therefore, investigated the potential role of small n and chance by comparing the observed mortality counts in Roseto to the expected counts had the underlying mortality rate been similar to Framingham, MA.

Diet

Dietary data for Roseto and the neighboring towns were limited. In Roseto, average daily calorie consumption was 3,000 for men and 2,300 for women [1], similar to that of other American adults. Calories were derived from fat (41%), protein (13%), carbohydrates (42%), and alcohol (5%). There were no data provided by Wolf and colleagues on the types of fats (saturated, mono- and poly-unsaturated, trans) or carbohydrates (refined versus unrefined) that were used by individuals in Roseto or neighboring towns, typical of studies in that era. The Rosetans’ high saturated fat intake was supported only by anecdotal observations. Favorite dishes included peppers fried in lard, and their bread was “dipped … in the lard gravy.” Prosciutto with “rim of fat, more than an inch thick” was commonly consumed [1,9].

The Rosetan diet likely included few ultra-processed foods. Lard was used in Italy for millennia. Rosetans cooked with olive oil (57%), lard, and butter (47%), while in Bangor, lard was used by 16% and butter by 11% [1]. Lard fat is 45% monounsaturated, 39% saturated (mainly palmitic and stearic acids), and polyunsaturated (11%). Palmitic acid, the main saturated fat in lard, comprises ~25% of fatty acids in our bodies. When dietary intake is high, our bodies synthesize less. Stearic acid is primarily metabolized to the monounsaturated oleic acid. Thus, ~70% lard fat is metabolized into mono- or polyunsaturated forms. Saturated fats increase the lighter and fluffier LDL fraction, which is not associated with heart disease risk, unlike the small dense LDL fraction, which is associated with heart disease risk [7]. Saturated fats also raise HDL, which is associated with lower heart disease risk. We lack data on the saturated fat consumption of Rosetans or residents of neighboring towns.

During the 1950s and 1960s, Crisco cooking oil was commonly used in American homes and contained trans and other synthetic fats. Internationally, the rise of CVD and other noncommunicable diseases (NCDs; e.g., obesity, diabetes, cancer, gout) is strongly linked to highly processed diets, as well as increased caloric intake. Consumption of sugar and other refined carbohydrates, potential causes of NCDs [14], were not studied in Roseto [1] nor in the Seven Countries Study [4]. Consumption of Crisco oil and other trans fats, as well as refined carbohydrates, may have differed between Roseto and the neighboring towns and contributed to differences in NCDs.

Genetics

The Rosetans’ ancestors came from Roseto Valforte, a small town in southeastern Italy, suggesting possible genetic factors. Genetic and lifestyle factors are independently associated with coronary disease [7]. Genetic enclaves may have increased or decreased coronary disease risk [7,9]. However, no evidence supported or refuted genetic factors in Rosetans’ heart disease. Wolf and colleagues argued that immigrants from Roseto Valforte who settled in other American cities had higher rates of heart disease, but no evidence supported this claim. Wolf and colleagues later reported that as Rosetans assimilated into American culture, their rates of heart disease rose [8]. Thus, genetic factors, never systematically studied, were unlikely to be a major factor.

Comorbid Disorders and Risk Factors

Data on CVD risk factors such as obesity, smoking, hypertension, and hypercholesterolemia in Roseto and neighboring towns were limited. While obesity was more prevalent among Rosetans than in neighboring towns, diabetes prevalence in Roseto was far lower than in Nazareth or Bangor [9]. As diabetes increases CVD and cardiovascular mortality by more than 50%, this could contribute to the relatively better cardiac health of Rosetans [15]. Rates of smoking were similar in Roseto and Bangor, although in neighboring towns, “the number of cigarettes smoked is somewhat higher than it is in Roseto” [1,10]. Among men, hypertension (>=150/90) was more common in Roseto than in Nazareth or Bangor, but rates in women were similar. Men’s mental health disorders were 50-300% lower in Roseto (162/100,000) compared to Bangor (289/100,000) and Nazareth (506 per 100,000) [9].

Did Neighboring Towns Have High Rates of CVD?

Rosetans’ heart health was compared to that of four neighboring towns. We assessed the incidence of deaths due to CVD in Roseto relative to the age-adjusted rates for men over 45 years of age in the Framingham study (see Table 1). Men in Bangor over 45 years had >fourfold the average CVD mortality (251/100,000)1 compared to similar-aged men in Framingham (Table 1). The Rosetan men’s heart health may partly reflect that Bangor was a ‘sick’ comparator group.

Wolf and colleagues combined MI deaths from autopsy-proven, clinically diagnosed, and “presumed,” but did not provide data on each category. No diagnostic criteria were specified for the ‘presumed’ group. Given the a priori belief that Rosetans had low rates of MI deaths, which instigated Wolf et al’s study, the lack of blinding may have led to more liberal diagnosing of presumed CVD deaths outside Roseto.

Outmigration and Birthplace

A paradox was the low percentage of older adult men in Roseto (8% ages 55-64 and 9% >65 years old) [1], lower than any of the four neighboring towns. Mortality rates in Roseto were reportedly low. Did Rosetan adult men leave after childhood or young adulthood, or did they die prematurely? The lack of data on the ‘missing men’ is a major limitation. Perhaps men who lived less healthy lifestyles and were less connected to the social community migrated out more frequently or died before Wolf et al’s study? Older Rosetan men included a higher percentage of Italian-born individuals, whose early childhood lifestyle and diet were another confounding factor, and would be predicted to be associated with lower CVD mortality.

Role of Statistical Uncertainty

A striking finding was the lack of any CVD deaths among 55-64-year-old men in Roseto versus a ~1% annual CVD mortality in neighboring towns. CVD deaths were also uncommon among men over the age of 65. However, in Roseto, only 62 men were aged 55-64, and 70 were over 65 years old. By contrast, in neighboring towns, the number (percentage) of men ages 55-64 and >65 in Bangor (400 (12%) and 400 (12%)), Stroudsburg (329 (12%) and 412 (15%)), East Stroudsburg (332 (9%) and 332 (9%)), and Nazareth (328 (11%) and 328 (11%)) were higher. The Roseto data for men >55 years old were limited by small numbers (132 men were 55 years or older). Whenever sample sizes are small, people intuitively believe observations (e.g., low CVD rates) can be generalized, but this is a fallacy [16].

## Discussion

Our analysis shows that the overall CVD mortality rate in Roseto, PA, was similar to that in Framingham, MA, during the 1950s. Thus, there may never have been a Roseto effect, and therefore neither social support, genetics, diet, nor other risk factors should be invoked to explain Roseto’s apparent immunity to CVD disease and death. The Roseto effect was originally interpreted to support that stress and social support prevented heart disease, while the Diet-Heart Hypothesis considered that total dietary fats and saturated animal fats caused heart disease. Both conclusions were fraught with potential selection and confounding biases. Heart disease was considered epidemic in the early 1950s, but the apparent rise was largely attributable to an aging population, increased ECG use, revised diagnostic MI criteria, and 1949 revised cause-of-death criteria [17]. The latter increased cardiac deaths by 20-35% in one year [18]. AHA created a media campaign to promote the need for research funding. In 1953, Keys linked rising cholesterol levels to the 26.4% increase in fat consumption by Americans between 1909 and 1952 [19]. This increase was primarily ‘cooking fats and oils’ excluding butter [19,20], and in 1953 Keys recommended reducing cooking fats and oils. Notably, lard, butter, and whole milk consumption declined >50% from 1900 to 1960 [20]. However, the first AHA dietary recommendations advocated reducing saturated as well as total fats [5]. He modified the changing American diet narrative from rising vegetable oils, documented in USDA data [19], to rising animal fat consumption, which USDA data showed had declined [20].

Roseto became a model for the idea that inter-generational and community social support protects against CVD and prolongs life. However, direct comparison of rates of CVD deaths without accounting for clinical, environmental, and lifestyle confounders can create selection bias. Although genetics and lifestyles likely differed between residents of Roseto, PA, and Framingham, MA, we used Framingham as the best contemporaneously studied American population for CVD. The original FHS cohort was of European descent and free of CVD at enrollment. Our hypothesis that lower male CVD mortality in Roseto may reflect small population size and chance was supported by our analysis. Compared to FHS, observed MI death rates in Roseto were similar, except for subjects over age 65, where Roseto had a higher MI death incidence.

The flawed interpretation of the Roseto data resulted from methodological issues. Wolf et al. did not assess a random selection of small towns. He was told Roseto had a low MI death rate, introducing the “sharpshooter bias” [9,10]. He committed resources to a non-blinded study limited by small numbers and had limitations in the methods of data collection and what data was collected. Roseto’s politicians and residents collected data and sought to promote their town favorably. Data sampling and interpretation were likely suffering from confounding biases. For example, many members of Roseto were in charge of collecting data and revealed that they aimed to present their community in a favorable light [21]. Confounding variables, such as far higher rates of diabetes (e.g., 26/1,000 men and 37/1,000 women in Roseto; 44 and 94/1,000 for men and 30 and 102/1,000 for women in Nazareth and Bangor, respectively) and mental health disorders (162/100,000 men and 234/100,000 women in Roseto; 505/100,000 men and 518/100,000 women in Nazareth; 289/100,000 men and 351/100,000 women in Bangor) in neighboring towns [22], were not considered. Demographic, anthropometric, dietary, medical, and cause-of-death data for Roseto and “control” towns were scant. Social life was qualitatively assessed in Roseto only. Mortality data on men who left Roseto, used to refute potential genetic factors, were not presented. The paucity of Rosetan men over 55 conflicts with the narrative of healthy older men bonded to their community by potent intergenerational ties. The early life environment in Italy was another confounding factor. Data on comorbid disorders and risk factors, including diet, in comparison with towns, are critical to evaluate potential causal factors, such as social support. While neighboring towns had relatively higher rates of cardiac deaths compared to Roseto, such deaths in Roseto were similar to or higher than those in Framingham, MA, over the same period.

A key lesson from the Roseto study is that biases are powerful, pervasive, and persistent. The Roseto study never supported that social support improves heart health with solid evidence, yet it continues to be cited in medical [23], best-selling nonfiction books [12], and on Wikipedia [24]. Promoted to refute that saturated fats cause heart disease, its data on diet and disease were inadequate. Similarly, the Diet-Heart hypothesis has also been criticized due to correlative data that were plausible but unproven [25]. Both the Roseto and Diet-Heart Hypotheses, as well as Keys’ earlier total fat-heart disease hypothesis, were confounded by selection bias [25,26] and uncontrolled variables (e.g., sugar and other highly processed foods) [20]. The Diet-Heart Hypothesis was not supported by regional, national, and international studies [25,27], as well as prospective observational and randomized controlled trials [25,28]. Further, although Keys used the rising consumption of all fats from 1909 to 1952 in America to implicate dietary fats in the rapid rise of heart disease, only polyunsaturated fat consumption rose dramatically during the first half of the 20th century, while saturated fat consumption declined during this period. Dietary trials in high-risk-for-CVD individuals that replaced saturated fats with vegetable oils showed heart disease deaths declined, but overall mortality was unchanged [7,27,28]. The Framingham Heart Study Diet Study revealed that an individual’s dietary fat or saturated fat consumption did not correlate with their serum cholesterol or risk of heart disease [29]. However, publication of this study was suppressed [30]. Although the role of saturated fats in heart health remains controversial, belief without definite evidence characterized both the Roseto thesis and the Diet-Heart Hypothesis.

In most nutritional science, dozens of variables were imprecisely measured or not assessed. Many of these limitations persist today. For example, long-term assessment of total daily calories, amount and type of macronutrients (e.g., sugar vs. unrefined carbohydrates, saturated vs. polyunsaturated fats) is confounded by recall, hindsight, quantification, illusion of validity, and other biases. Further, these potential limitations are compounded because diets are sampled at a limited number of times, while the potential pathogenic effects of diet extend across decades. Limitations of our comparison of the Roseto study to the FHS include different (1) geographies in the northeastern United States (Eastern Pennsylvania and Central-Eastern Massachusetts), (2) different ethnicities (Roseto: Italian; Framingham: Italian, Irish, and English), and the studies had different (3) designs (Roseto: retrospective; Framingham: prospective), and (4) criteria to diagnosis MI and MI-related mortality.

Studies where variables over long durations cannot be accurately assessed, as in nutrition and medical outcomes, are liable to the narrative fallacy, where a logical explanation makes them more cohesive and collectively compelling. When definitive data are elusive, when a theory is accepted by a small group, its success may relate more to the conviction of protagonists and contagion - how many times references are cited and the theory dominates high-impact scientific and lay articles - than to its veracity. In most nutritional science, dozens of variables were imprecisely measured or ignored. Many of these limitations persist today. Notoriously difficult and unreliable measures include total daily calories, amount and type of macronutrients (e.g., sugar v. unrefined carbohydrates, saturated v. polyunsaturated fats). Further, these potential limitations are compounded because diets are sampled at one time while the pathogenic effects of diet develop over decades. The Roseto and Diet-Heart Hypotheses exemplify the vulnerability of science where precise data is lacking, confounded factors abound, and biases are fueled by authority and contagion.

A fundamental limit of science and medicine is the illusion that its current knowledge is correct or that its hypotheses incorporate the range of possible explanations. The Roseto Study challenged the emerging consensus in the early 1960s that fat and saturated fat consumption were primary drivers of heart disease. In hindsight, this focus was likely misdirected, and yet the Roseto Study data did refute this data. Rather, rates of heart disease in this community were similar to or higher than those in the one population with higher quality prospectively collected data. Our study also highlights the challenges of studies that assess the long-term outcomes based on diet. Given the large number of variables in diet, changes over years and decades, other genetic and environmental factors that can only be partially quantified, conclusions based on dietary studies should be tentative. When such studies are conducted over short periods of time or in artificial settings, results cannot be extrapolated to long-term outcomes in other populations. Neither the Diet-Heart Hypothesis nor the Social Support Hypothesis is now considered to be the major cause of heart disease. Although diet does strongly influence heart health, other factors, such as sugar and other refined carbohydrates and total caloric intake, as well as many other factors, such as physical activity, blood pressure, inflammation, and sleep, also influence heart health. While social support can contribute to health outcomes, the broader social determinants of health, including the conditions where people are born, grow, work, live, and age, and the forces and systems that shape their daily lives, are now considered a major factor in health outcomes.

The limits of the Diet-Heart and Social Support Hypotheses are relevant to current medicine. We are liable to the narrative fallacy, where a logical explanation can weave information into a cohesive fabric, which makes it collectively compelling and explanatory. When definitive data are elusive, theories may be accepted by a small group, and the success of the theory may relate more to the conviction of protagonists and contagion - how many times references are cited and the theory dominates high-impact scientific and lay articles - rather than scientific merit. In most nutritional science, dozens of variables were imprecisely measured or ignored. These limitations persist to this day. Total daily calories, amount and type of macronutrients (e.g., sugar vs. unrefined carbohydrates, saturated vs. polyunsaturated fats) are difficult to measure accurately over time. The Roseto and Diet-Heart Hypotheses exemplify the vulnerability of science where precise data is lacking, confounded factors abound, and biases are fueled by authority and contagion. In the debate between these two hypotheses, alternative hypotheses were rarely considered. 

## Conclusions

The Roseto study posed a critical challenge to the Diet-Heart Hypothesis, as this hypothesis was transitioning from a theory toward established dogma. Ancel Keys, father of the Diet-Heart Hypothesis, launched a vigorous defense of his theory by identifying valid weaknesses of the Roseto study. However, many of these weaknesses extended to the Diet-Heart Hypothesis, which is now accepted by leading health agencies as fact. Stewart Wolf, lead investigator for the Roseto study, hypothesized that social cohesion was the basis for the extraordinarily positive heart health outcomes of Rosetans, especially older men. However, the small sample, when assessed statistically against the only United States population with prospective observational data, in Framingham, MA, revealed that the residents in Roseto had expected higher rates of MI deaths. Our study highlights the limits of epidemiology, especially when data is collected retrospectively to assess a favored hypothesis.
