# Correction: The Roseto Study: Selection Bias Versus Social Support

**DOI:** 10.7759/cureus.c467

**Published:** 2026-08-05

**Authors:** Samrachana Adhikari, Olugbenga G Ogedegbe, Orrin Devinsky

**Affiliations:** 1 Department of Population Health, NYU Grossman School of Medicine, New York, USA; 2 Institute for Excellence in Health Equity, NYU Grossman School of Medicine, New York, USA; 3 Department of Neurology, NYU Grossman School of Medicine, New York, USA

This article has been corrected to update the following author affiliations:

Samrachana Adhikari: Department of Population Health, NYU Grossman School of Medicine, New York, USA

Olugbenga Ogedegbe: Institute for Excellence in Health Equity, NYU Grossman School of Medicine, New York, USA

Orrin Devinsky: Department of Neurology, NYU Grossman School of Medicine, New York, USA

